# Supplementary material for: Aroma Identification and Classification in 18 Kinds of Teas (Camellia sinensis) by Sensory Evaluation, HS-SPME-GC-IMS/GC × GC-MS, and Chemometrics
Source: Foods. 2023 Jun 21;12(13):2433. doi: 10.3390/foods12132433 (PMC10340347; doi:10.3390/foods12132433)
Supplement: Supplementary file 1 [file foods-12-02433-s001.zip › foods-2455812-supplementary.pdf]

Table S1. The result of the aroma profile evaluation

tea samples	Score of each aroma attribute							
	chestnut-like	fresh	floral	fruity	sweety	fermented aroma	roasty	burning
NO.1	8	9	6	3	2	0	1	0
NO.2	5	9	8	3	1	0	2	0
NO.3	3	9	3	0	0	0	1	0
NO.4	6	9	5	2	0	0	3	0
NO.5	3	9	4	0	0	0	1	0
NO.6	5	9	6	1	0	0	2	0
NO.7	7	8	7	1	2	0	5	0
NO.8	5	9	5	1	1	0	4	0
NO.9	3	9	6	0	0	0	2	0
NO.10	1	5	9	9	7	4	8	0
NO.11	1	6	9	7	6	3	6	0
NO.12	1	8	9	6	5	3	5	0
NO.13	1	8	9	6	6	3	7	0
NO.14	1	5	9	8	7	4	8	0
NO.15	0	3	7	7	9	5	5	0
NO.16	0	3	8	7	9	4	5	0
NO.17	0	1	8	6	9	6	6	0
NO.18	0	0	3	5	8	5	7	8

Table S2. the qualitative results of 18 kinds of teas by HS-GC-IMS

Count	Compounds [1]	Formula	MW	RI [2]	Rt [sec] [3]	Dt [RIPrel] [4]
1	propionaldehyde	C3H6O	58.1	815	244.408	1.1459
2	acetone	C3H6O	58.1	836	253.842	1.1168
3	acrolein	C3H4O	56.1	872	271.695	1.062
4	ethyl acetate	C4H8O2	88.1	902	288.81	1.3394
5	2,5-dimethylfuran	C6H8O	96.1	911	294.418	1.0274
6	2-butanone	C4H8O	72.1	916	297.569	1.2471
7	3-methylbutanal	C5H10O	86.1	930	307.349	1.402
8	ethanol	C2H6O	46.1	946	318.006	1.0415
9	2-ethylfuran	C6H8O	96.1	962	330.116	1.0469
10	2,3-butanedione	C4H6O2	86.1	974	340.028	1.165
11	pentanal	C5H10O	86.1	992	354.88	1.4258
12	ethyl acrylate	C5H8O2	100.1	1017	377.782	1.1265
13	2,3-pentanedione	C5H8O2	100.1	1018	379.62	1.2238
14	ethyl 2-methylbutanoate	C7H14O2	130.2	1019	380.585	1.2333
15	ethyl 3-methylbutanoate	C7H14O2	130.2	1023	383.726	1.2567
16	propyl acetate M	C5H10O2	102.1	1025	386.176	1.1654
17	propyl acetate D	C5H10O2	102.1	1026	387.166	1.4781
18	ethyl propanoate	C5H10O2	102.1	1027	387.763	1.145
19	α-pinene	C10H16	136.2	1027	388.409	1.2163
20	1-propanol	C3H8O	60.1	1040	401.381	1.1083
21	ethyl butanoate M	C6H12O2	116.2	1048	409.833	1.2061
22	ethyl butanoate D	C6H12O2	116.2	1047	409.264	1.5602
23	hexanal	C6H12O	100.2	1093	463.902	1.5647

24	2-methylpropanol	C4H10O	74.1	1102	475.837	1.1674
25	β-pinene	C10H16	136.2	1111	488.409	1.2191
26	butyl propionate	C7H14O2	130.2	1134	523.595	1.2806
27	butanol M	C4H10O	74.1	1157	559.819	1.1835
28	butanol D	C4H10O	74.1	1157	560.347	1.3788
29	2-methylbutanol	C5H12O	88.1	1170	584.007	1.2328
30	pentyl acetate	C7H14O2	130.2	1188	617.101	1.3121
31	ethyl valerate	C7H14O2	130.2	1192	623.905	1.2635
32	2-heptanone	C7H14O	114.2	1197	633.374	1.6304
33	heptanal	C7H14O	114.2	1204	648.026	1.3284
34	limonene M	C10H16	136.2	1208	657.706	1.2196
35	limonene D	C10H16	136.2	1209	658.563	1.291
36	limonene T	C10H16	136.2	1208	657.283	1.656
37	3-methylbutanol M	C5H12O	88.1	1223	688.846	1.243
38	3-methylbutanol D	C5H12O	88.1	1222	687.023	1.4888
39	o-xylene	C8H10	106.2	1226	694.721	1.0662
40	ethyl caproate	C8H16O2	144.2	1233	712.352	1.3403
41	2-pentylfuran	C9H14O	138.2	1246	742.491	1.2526
42	2-hexanol	C6H14O	102.2	1248	746.443	1.2807
43	cyclohexanone	C6H10O	98.1	1259	774.362	1.1493
44	beta-ocimene	C10H16	136.2	1262	783.593	1.2147
45	1-pentanol M	C5H12O	88.1	1266	793.463	1.2526
46	1-pentanol D	C5H12O	88.1	1267	794.98	1.5089
47	(Z)-3-hexenyl acetate	C8H14O2	142.2	1268	799.496	1.3039
48	2-methylpyrazine M	C5H6N2	94.1	1279	827.85	1.073
49	2-methylpyrazine D	C5H6N2	94.1	1279	827.75	1.0882
50	2-octanone	C8H16O	128.2	1297	880.987	1.3343
51	octanal M	C8H16O	128.2	1301	891.341	1.3998
52	octanal D	C8H16O	128.2	1300	889.701	1.8242
53	1-hydroxy-2-propanone	C3H6O2	74.1	1306	906.463	1.0421
54	(E)-3-hexenol	C6H12O	100.2	1311	923.089	1.2369
55	3-methylpentanol	C6H14O	102.2	1318	947.643	1.3111
56	2,5-dimethylpyrazine	C6H8N2	108.1	1327	975.184	1.1139
57	(E)-2-heptenal	C7H12O	112.2	1332	992.725	1.2568
58	methyl heptenone	C8H14O	126.2	1347	1046.964	1.1772
59	dimethyl trisulfide	C2H6S3	126.3	1357	1081.685	1.3042
60	1-hexanol M	C6H14O	102.2	1367	1122.538	1.3221
61	1-hexanol D	C6H14O	102.2	1366	1117.489	1.6379
62	ethyl lactate	C5H10O3	118.1	1381	1179.318	1.1433
63	(Z)-3-hexenol M	C6H12O	100.2	1393	1233.068	1.2291
64	(Z)-3-hexenol D	C6H12O	100.2	1394	1234.446	1.5094
65	nonanal	C9H18O	142.2	1398	1251.23	1.4721
66	2-cyclohexenone	C6H8O	96.1	1404	1279.452	1.1149
67	2-butoxyethanol	C6H14O2	118.2	1407	1295.776	1.2919
68	(E)-2-octenal	C8H14O	126.2	1427	1392.429	1.3346
69	trimethylpyrazine	C7H10N2	122.2	1433	1421.013	1.1679
70	acetic acid	C2H4O2	60.1	1454	1532.292	1.0509
71	1-octen-3-ol	C8H16O	128.2	1464	1585.289	1.1626
72	diallyl disulfide M	C6H10S2	146.3	1465	1592.656	1.1991
73	diallyl disulfide D	C6H10S2	146.3	1466	1596.792	1.632
74	ethyl octanoate	C10H20O2	172.3	1467	1601.005	1.4761

75	furfural M	C5H4O2	96.1	1468	1609.8	1.0837
76	furfural D	C5H4O2	96.1	1468	1607.942	1.3346
77	(E,E)-2,4-heptadienal M	C7H10O	110.2	1487	1721.241	1.1879
78	(E,E)-2,4-heptadienal D	C7H10O	110.2	1487	1720.072	1.6209
79	2-acetylfuran	C6H6O2	110.1	1502	1815.286	1.1147
80	(E)-2-nonenal	C9H16O	140.2	1502	1818.222	1.4072
81	benzaldehyde M	C7H6O	106.1	1508	1858.176	1.1509
82	benzaldehyde D	C7H6O	106.1	1509	1859.845	1.4716
83	bornyl acetate	C12H20O2	196.3	1565	2276.104	1.2187
84	5-methylfurfural	C6H6O2	110.1	1568	2302.57	1.1253
85	methyl benzoate	C8H8O2	136.1	1575	2357.601	1.2025

1. The voltile compounds detected in all 18 kinds of teas by GC-IMS. The signal M represented monome, the signal D represented dimer, and the T represented Trimer.

2. RI, retention index.

3. RT, retention time.

4. DT, relative migration time.

Table S3. The aroma compounds in 18 kinds of teas detected by GC×GC-MS

no.	RI	aorma compounds	identification methods	perception	peak areas of tea samples																	
					NO.1	NO.2	NO.3	NO.4	NO.5	NO.6	NO.7	NO.8	NO.9	NO.10	NO.11	NO.12	NO.13	NO.14	NO.15	NO.16	NO.17	NO.18
1	813	2-methylpropanal	MS/RI/S	herbal green	1591.94	0.00	3.22	275.36	105.92	495.28	2064.99	186.94	838.85	0.00	850.65	129.05	1005.22	1204.56	1750.82	2866.93	745.62	1765.12
2	814	acetone	MS/RI	solvent 、 fruit	252.21	103.02	293.89	17.18	81.79	47.77	777.72	79.11	325.30	340.62	113.16	31.49	0.00	228.36	41.68	63.24	0.00	273.10
3	832	3-methylfuran	MS/RI	chocolate	0.00	0.00	959.66	107.38	73.10	13.65	0.00	133.60	369.92	0.00	228.48	155.61	180.22	0.00	0.00	959.06	0.00	566.59
4	864	methyl acetate	MS/RI	green, etherial, fruity, fresh, rum and whiskey-like	944.46	0.00	154.64	0.00	0.00	1.10	708.94	0.00	252.73	1805.29	337.70	0.00	454.80	2152.75	0.00	443.47	194.35	305.98
5	876	2-methylfuran	MS/RI	chocolate	630.31	0.00	945.36	505.65	449.45	540.32	1306.35	436.98	247.98	1560.52	1730.51	401.71	1520.15	2822.33	55.64	60.22	104.10	1375.48
6	907	ethyl acetate	MS/RI/S/O	sweet waxy floral	2124.63	0.00	749.95	533.34	462.50	873.25	804.07	326.91	638.93	9543.84	173.31	316.66	241.90	0.00	764.25	0.00	264.71	208.23
7	910	3-methylbutanal	MS/RI/S/O	fruity nutty cocoa	0.00	138.27	0.00	1082.47	2357.28	10.22	871.49	90.34	3174.00	0.00	2161.91	658.87	6088.07	72.69	7931.99	8120.79	5214.58	0.00
8	912	2-methylbutanal	MS/RI/S/O	cocoa, almond	9147.84	0.00	1138.19	1335.77	0.00	3628.03	14034.04	6232.86	3715.54	4033.55	2224.08	0.00	0.00	8507.91	7125.03	10995.98	3708.87	13765.76
9	935	pentanal	MS/RI/S/O	nutty、 cocoa	4188.38	792.40	3156.80	3137.76	6437.30	4805.61	8300.61	9177.41	9755.67	2667.50	5947.33	5098.19	9433.92	4258.44	1237.99	4601.33	3990.48	5506.36
10	955	2-ethylfuran	MS/RI	solvent ethereal brown cocoa beany rooty earthy musty	808.85	0.00	1568.94	1443.63	316.89	1188.28	1664.53	286.09	868.00	3433.33	1496.22	532.34	2076.64	5361.72	767.39	3451.10	2112.62	5859.01
11	973	1-penten-3-one	MS/RI/S/O	fish, pungent	520.29	62.40	8077.05	376.74	249.83	295.19	989.61	436.00	324.06	2049.04	4287.90	3843.34	5690.77	3061.87	528.85	965.31	2110.08	1229.99
12	998	2,3-butanedione	MS/RI/S/O	creamy buttery	0.00	0.00	0.00	83.38	86.05	14.09	303.72	76.67	0.00	80.57	7.67	0.00	298.04	209.74	113.61	0.00	3.42	0.00
13	1000	amylene hydrate	MS/RI	pungent	0.00	66.74	0.00	0.00	0.00	43.29	0.00	0.00	25.00	103.29	0.00	0.00	0.00	409.50	27.80	235.52	148.81	202.52
14	1008	2-methyl-3-buten-2-ol	MS/RI/S	herbal earthy oily	0.00	0.00	36.75	29.04	0.00	0.00	0.00	0.00	0.00	0.00	0.00	15.63	0.00	0.00	0.00	9.87	15.92	34.89
15	1042	toluene	MS/RI/S	sweet	4579.93	1618.49	8015.37	2061.97	2264.03	1100.55	5816.12	3515.61	5214.73	13558.76	2674.57	4171.87	4245.35	17541.28	8032.06	4869.77	4775.10	2389.39
16	1047	(E)-2-butenal	MS/RI/S	flower	0.00	50.04	0.00	394.82	0.00	172.09	0.00	0.00	23.42	0.00	0.00	410.20	0.00	0.00	0.00	0.00	0.00	0.00
17	1054	2,3-pentanedione	MS/RI/S/O	cream, butter	309.00	0.00	412.04	69.01	42.74	141.73	778.06	100.03	356.93	210.74	274.54	156.38	977.97	455.95	61.80	138.08	177.03	94.60
18	1071	dimethyl disulfide	MS/RI/S/O	onion, cabbage	36.83	0.00	0.00	39.53	41.06	37.07	66.05	45.73	71.58	0.00	5.18	0.00	0.00	0.00	43.07	251.38	25.63	89.81
19	1084	hexanal	MS/RI/S/O	grass, fat	7207.50	1323.65	13862.30	9140.65	8010.29	15869.62	14519.28	5754.07	12764.01	20403.47	26911.82	15671.39	20868.99	24621.05	10443.62	18252.52	34163.38	36880.96
20	1090	pentanoic acid	MS/RI/S	sweat	0.00	0.00	0.00	0.00	0.00	6.60	0.00	0.00	103.21	149.80	0.00	148.30	199.32	282.42	73.97	244.83	266.85	431.76
21	1093	2-methylthiophene	MS/RI/S/O	sulfurous roasted green cabbage	0.00	0.00	0.00	0.00	0.00	0.00	0.00	0.00	0.00	270.79	0.00	0.00	0.00	0.00	0.00	0.00	0.00	0.00

				onion bitter																		
22	1101	2-methyl-2-butenal	MS/RI/S/O	green, fruit	139.55	0.00	601.12	77.82	56.40	64.66	195.13	41.46	138.28	1261.94	669.63	85.62	288.45	1295.11	670.47	1107.81	258.97	972.85
23	1131	(E)-2-pentenal	MS/RI/S/O	fruit	70.19	251.07	15797.86	1127.24	0.00	854.64	1386.47	601.92	27.22	4505.37	4945.83	4725.50	7304.76	5356.29	917.93	2170.11	3302.51	5639.49
24	1131	3-heptanone	MS/RI/S/O	green, fatty, fruity	2730.21	0.00	874.63	313.06	600.28	1598.27	1790.13	735.01	2426.63	208.62	112.55	271.86	306.90	413.74	2158.48	824.69	1953.89	216.31
25	1132	propanoic acid, butyl ester	MS/RI/S/O	fruity, sweet	0.00	0.00	0.00	55.46	109.19	0.00	104.69	87.81	117.10	0.00	0.00	0.00	0.00	59.40	281.44	0.00	231.73	0.00
26	1157	2-methyl-2-pentenal	MS/RI/S/O	sweet, fruity	0.00	0.00	550.45	342.42	160.13	243.75	601.34	49.02	755.98	892.27	860.07	119.54	485.09	1066.91	0.00	1681.84	390.13	2981.97
27	1157	1-penten-3-ol	MS/RI/S/O	butter, pungent	395.08	29.12	4422.71	810.65	96.06	905.33	2127.17	484.87	986.88	2241.93	2444.08	2716.20	4063.18	3717.53	284.02	1387.67	1654.32	1771.76
28	1158	trans-2-butenoic acid ethyl ester	MS/RI/S/O	musty, onion, garlic, caramellic	25.47	0.00	305.18	0.00	22.45	130.71	149.40	18.30	147.47	0.00	0.00	0.00	0.00	0.00	160.31	0.00	130.25	0.00
29	1166	α-phellandrene	MS/RI/S/O	turpentine, mint, spice	1317.18	0.00	148.23	0.00	0.00	211.78	327.32	42.60	221.13	812.77	78.20	226.81	442.29	570.77	3.20	1592.27	0.00	0.00
30	1170	β-myrcene	MS/RI/S/O	woody, citrus	6263.06	3389.08	1416.09	714.37	507.45	12327.35	3592.54	512.48	15696.59	6160.49	612.72	3147.44	4579.73	3788.24	17909.59	21053.70	12866.09	35690.32
31	1174	heptanal	MS/RI/S/O	fat, citrus	2424.10	718.55	8029.16	2588.24	2506.46	4741.69	5322.83	1464.02	3936.98	0.00	139.22	2984.24	9626.58	452.46	3063.08	3374.04	12857.53	8657.79
32	1177	methyl hexanoate	MS/RI/S/O	tropical fruit, creamy	0.00	0.00	0.00	0.00	0.00	0.00	239.63	0.00	175.35	2078.68	0.00	0.00	0.00	4453.11	178.69	1790.64	0.00	3207.41
33	1178	α-terpinene	MS/RI/S	lemon	1790.97	0.00	122.52	29.67	41.71	915.16	366.50	63.68	622.45	1720.34	94.48	709.65	411.34	1141.27	0.00	1366.94	595.25	1608.07
34	1178	1-ethyl-1H-pyrrole	MS/RI/S/O	burnt	16305.14	0.00	199.40	1384.14	603.16	382.97	5895.79	617.64	3255.09	6044.92	881.85	99.05	482.44	7189.33	754.98	516.67	107.57	353.92
35	1201	(+)-limonene	MS/RI/S/O	citrus	9108.46	5843.43	3323.41	1334.69	3008.01	51365.27	9801.81	3332.28	10219.72	16160.53	0.00	86821.33	9386.96	15783.55	20459.12	12707.34	11880.76	9962.56
36	1201	(E)-2-hexenal	MS/RI/S/O	apple, green	589.58	218.42	16127.13	985.31	199.71	2800.75	610.93	258.71	0.00	6633.77	3016.42	1303.65	2001.18	7797.39	12577.65	11491.75	20054.69	30916.54
37	1206	3-methyl-2-butenal	MS/RI/S/O	sweet, fruity, green	140.36	0.00	647.38	192.41	0.00	88.48	91.47	290.25	62.82	381.54	324.26	254.42	140.45	300.28	104.38	215.04	2126.92	388.49
38	1209	β-phellandrene	MS/RI/S/O	mint, terpentine	1713.96	119.32	0.00	33.94	18.03	0.00	458.02	35.90	270.16	893.38	121.50	0.00	0.00	0.00	0.00	1722.10	1012.66	0.00
39	1209	pyrazine	MS/RI/S	roasted hazel nut	0.00	0.00	0.00	0.00	0.00	0.00	0.00	0.00	0.00	0.00	0.00	0.00	0.00	436.83	135.67	0.00	0.00	149.06
40	1213	1,8-cineole	MS/RI/S	mint	0.00	0.00	557.25	76.08	28.78	202.62	197.20	70.10	334.47	0.00	0.00	0.00	0.00	0.00	173.89	117.16	0.00	0.00
41	1220	ethyl hexanoate	MS/RI/S	apple peel, fruit	0.00	0.00	348.42	102.89	174.64	467.47	846.72	166.04	971.17	93.09	27.42	89.01	157.16	111.24	802.16	763.39	933.29	383.97
42	1225	butanoic acid, butyl ester	MS/RI/S	sweet, fruity, fatty	135.88	0.00	0.00	78.63	169.56	147.41	456.99	189.98	433.30	0.00	90.88	0.00	0.00	0.00	1002.09	252.50	762.61	0.00
43	1230	4-heptenal	MS/RI/S/O	biscuit, cream	931.31	69.21	12033.39	597.18	164.72	535.82	1247.82	364.16	304.38	3576.66	1983.50	2927.11	5233.97	6062.87	505.55	1172.37	3761.46	1964.21
44	1236	6-methyl-2-heptanone	MS/RI/S/O	camphoreous	0.00	28.85	182.21	401.54	0.00	343.99	260.96	18.38	184.53	1321.45	139.97	78.36	322.96	1540.09	279.30	460.47	548.67	796.22
45	1238	γ-terpinene	MS/RI	citrus, lime-iike	1197.24	121.16	194.07	121.65	152.63	5332.30	531.08	137.20	754.97	946.15	136.27	8367.72	910.68	935.72	1668.70	1212.81	759.36	1148.37

46	1240	2-pentylfuran	MS/RI/S/O	fruity, metallic	560.38	160.73	892.23	476.89	243.99	1541.62	1585.35	215.16	746.26	7571.23	833.45	875.77	5403.40	9412.96	1975.29	8081.50	6373.03	10424.37
47	1242	trans-β-ocimene	MS/RI	sweet, herb	2214.73	723.51	243.29	89.96	0.00	3392.52	787.33	0.00	4621.16	2192.26	0.00	430.87	1650.19	1568.14	0.00	7875.44	4157.04	0.00
48	1243	(E)-2-heptenal	MS/RI/S/O	soap, fatty	0.00	224.01	8168.60	0.00	0.00	0.00	0.00	0.00	0.00	0.00	91.15	0.00	0.00	0.00	0.00	0.00	0.00	6525.66
49	1248	prenyl acetate	MS/RI	sweet, banana, fruity, ripe	0.00	0.00	0.00	0.00	0.00	0.00	0.00	0.00	0.00	0.00	0.00	0.00	0.00	0.00	0.00	0.00	132.27	0.00
50	1251	ocimene	MS/RI/S/O	floral, green citrus, woody	2825.76	975.98	642.86	0.00	409.07	4974.65	1073.11	147.91	6088.38	2804.41	564.11	3762.31	9307.20	1783.03	15335.67	10751.45	5728.68	37077.94
51	1253	methyl (Z)-3-hexenoate	MS/RI/S/O	fruity floral	0.00	0.00	0.00	0.00	0.00	0.00	0.00	0.00	0.00	4971.66	0.00	0.00	0.00	4572.68	0.00	0.00	0.00	0.00
52	1255	1-pentanol	MS/RI/S/O	fusel, sweet	996.16	621.42	478.82	1904.27	1680.36	2798.50	6048.78	2878.58	4875.22	2109.92	3653.81	2394.60	4540.89	3530.85	600.93	3188.98	2854.81	2823.23
53	1258	cis-β-hexenyl formate	MS/RI/S/O	green, waxy, fresh vegetable, fruity, apple, guava, green banana	0.00	0.00	0.00	0.00	0.00	97.42	0.00	0.00	0.00	137.68	0.00	0.00	0.00	0.00	20.99	0.00	0.00	221.57
54	1264	methylpyrazine	MS/RI/S/O	nutty, roasted	591.30	235.98	0.00	34.37	0.00	11.00	550.80	11.46	300.96	3319.94	68.44	55.21	46.43	6599.04	501.45	1543.77	571.55	83.37
55	1280	hexyl acetate	MS/RI/S/O	fruity, green, fresh, sweet, banana peel, apple and pear	0.00	211.80	194.89	0.00	0.00	279.23	173.34	11.39	309.98	0.00	0.00	0.00	0.00	0.00	418.56	0.00	303.54	1268.32
56	1280	octanal	MS/RI/S/O	fat, soap,	1141.77	1909.86	2409.84	2390.47	1275.25	2217.45	2310.93	840.86	2054.31	4312.61	1171.79	1320.48	2250.68	2769.45	1574.72	2324.54	4348.15	6090.24
57	1284	methyl (2E)-2-hexenoate	MS/RI/S/O	fruity, green, banana, honey	0.00	0.00	0.00	0.00	0.00	0.00	0.00	0.00	0.00	2736.97	0.00	0.00	0.00	0.00	0.00	1442.92	0.00	0.00
58	1285	2-octanone	MS/RI/S	dairy,cheese,	206.35	0.00	15.27	18.96	0.00	194.98	0.00	48.03	0.00	1705.69	350.50	63.35	115.45	3157.34	311.02	1296.11	924.71	1589.27
59	1310	(Z)-2-penten-1-ol	MS/RI/S/O	green, spicy	246.01	281.30	6638.57	1023.95	0.00	1081.53	1868.35	297.82	1009.54	2617.39	2226.47	3231.79	3933.67	4569.43	888.32	2114.90	1785.97	2687.01
60	1313	1-octen-3-one	MS/RI/S/O	mushroom, metal	0.00	59.32	705.97	109.72	326.03	0.00	964.95	273.84	584.14	451.86	234.36	0.00	0.00	0.00	0.00	0.00	0.00	0.00
61	1315	cis-3-hexenyl-1-acetate	MS/RI/S	green fruity apple and pear, with fresh tropical nuances	83.66	0.00	3233.19	21.82	72.47	3699.97	0.00	26.34	66.36	618.07	0.00	159.98	72.96	0.00	0.00	0.00	0.00	2072.38
62	1321	trans-2-pentenol	MS/RI/S	mushroom	0.00	0.00	661.31	48.96	46.59	66.32	222.74	19.84	143.65	341.82	318.21	278.64	350.03	30.56	106.58	208.93	226.42	294.35
63	1323	2,5-dimethylpyrazine	MS/RI/S/O	cocoa, roasted nuts, roast beef, woody grass	0.00	138.02	0.00	0.00	0.00	0.00	3092.80	0.00	126.32	3667.41	0.00	0.00	0.00	8693.05	0.00	0.00	0.00	0.00

64	1328	2,6-dimethylpyrazine	MS/RI/S	meaty, nutty, roasted cocoa, brothy bready, coffee, musty	279.25	0.00	0.00	28.59	0.00	0.00	346.18	0.00	158.77	0.00	0.00	0.00	0.00	2915.24	98.78	176.54	206.94	0.00
65	1333	2-ethylpyrazine	MS/RI/S/O	nutty, musty, casky, woody, potato, earthy and cocoa with a fishy nuance	0.00	0.00	0.00	0.00	0.00	0.00	364.26	0.00	160.99	3537.22	0.00	0.00	0.00	4125.35	0.00	719.97	100.41	0.00
66	1335	2,2,6-trimethyl-cyclohexanone	MS/RI/S	honey cistus	796.38	184.74	905.10	536.28	160.93	841.79	1436.47	280.25	631.18	5894.43	1052.04	534.37	2676.50	10608.02	3927.27	3251.72	4254.31	4662.06
67	1336	6-methyl-5-hepten-2-one	MS/RI/S/O	green, vegetative	2496.76	444.15	11910.47	4570.40	971.97	3852.20	4931.18	757.05	2973.85	24699.44	8831.84	11216.09	9581.00	22187.32	5244.11	12798.81	9518.79	17369.67
68	1346	2, 3-dimethylpyrazine	MS/RI/S	nutty, cocoa, roasted coffee, bready	108.49	15.55	0.00	23.63	0.00	0.00	135.62	0.00	92.72	277.37	10.60	23.60	0.00	1281.22	87.00	268.36	161.24	0.00
69	1354.974287	(Z)-3-hexenyl-2-methylbutanoate	MS/RI/S/O	herb, sweet	141.55	0.00	403.88	25.27	74.05	3344.17	3956.62	21.43	3226.94	4037.11	220.29	1076.26	1003.89	1917.87	719.06	812.39	2036.61	15203.15
70	1360	1-hexanol	MS/RI/S/O	resin, flower	0.00	272.63	1683.30	408.02	0.00	4129.55	1748.73	153.10	2645.02	674.66	612.33	0.00	1385.62	0.00	7126.98	4396.98	2889.06	7149.59
71	1377	dimethyl trisulfide	MS/RI/S/O	sulfur, fish, cabbage	31.95	0.00	0.00	0.00	5.97	0.00	67.49	0.00	94.72	0.00	0.00	0.00	0.00	0.00	36.48	78.34	0.00	0.00
72	1385	nonanal	MS/RI/S/O	fat, citrus	12191.92	2390.95	10508.28	7889.29	18627.91	14908.53	10159.18	6053.24	10270.10	9323.33	2889.78	4550.35	5179.11	6057.19	8584.77	9103.27	14973.70	10714.92
73	1385	2-ethyl-6-methylpyrazine	MS/RI/S/O	roasted hazelnut	648.04	121.83	0.00	0.00	0.00	19.00	1169.14	0.00	162.29	2376.42	53.79	0.00	0.00	3496.99	0.00	442.02	0.00	0.00
74	1386	(Z)-3-hexen-1-ol	MS/RI/S/O	grass	364.55	768.94	7347.50	1628.34	1358.22	13397.06	6741.97	241.30	154.94	148.85	0.00	441.31	0.00	922.12	87.63	7923.37	7772.94	0.00
75	1392	2-ethyl-5-methylpyrazine	MS/RI/S/O	coffee bean, nutty, grassy, roasted	0.00	0.00	0.00	52.96	0.00	50.28	1810.51	0.00	802.52	3804.11	0.00	0.00	0.00	4901.84	0.00	1034.42	0.00	0.00
76	1394	1-octen-3-ol	MS/RI/S/O	mushroom	1965.69	429.86	3440.28	1747.77	1072.33	3738.20	9584.61	934.87	8744.19	5252.61	1806.46	1193.25	3109.49	6670.95	2944.96	5165.36	6561.57	6995.70
77	1397	2,4-hexadienal	MS/RI/S/O	sweet, green, waxy, aldehydic with fresh melon nuances	25.49	0.00	230.24	55.46	11.91	0.00	62.58	0.00	35.79	1052.32	301.71	179.90	62.13	838.57	283.65	454.30	854.29	2398.98
78	1401	(E,E)-2,4-heptadienal	MS/RI/S/O	nut, fat	5167.55	312.64	68818.40	2895.33	801.16	3398.47	6018.18	1175.34	1780.81	35557.95	15957.48	17430.98	22625.21	45926.57	3513.04	7815.02	12779.12	41283.57
79	1410	(E)-2-hexenol	MS/RI/S	green	0.00	30.54	1316.78	0.00	38.15	3514.37	760.40	19.72	1148.28	385.69	0.00	0.00	0.00	1680.17	4146.75	1189.04	26.15	2672.28
80	1414	3-octen-2-one	MS/RI/S/O	creamy	0.00	43.40	667.01	279.16	72.88	688.75	0.00	51.75	0.00	0.00	1182.23	300.23	551.32	0.00	0.00	2571.58	2667.89	8094.17
81	1414	2-ethyl-3-methylpyrazine	MS/RI/S/O	nutty peanut, musty corn-like with raw and oily	1275.77	21.25	0.00	0.00	0.00	0.00	691.62	0.00	306.27	4894.36	0.00	0.00	0.00	6800.97	0.00	0.00	0.00	0.00

				nuances																		
82	1420	acetic acid, 2-ethylhexyl ester	MS/RI/S/O	herbal	1193.86	0.00	2073.81	834.34	1154.70	5026.00	5080.67	996.04	4246.88	0.00	39.76	81.39	140.38	249.50	1151.50	900.45	743.49	1512.99
83	1420	(Z)-linalool oxide	MS/RI/S/O	flower	0.00	0.00	2606.83	0.00	0.00	11920.79	2315.97	84.56	3604.25	13997.84	4626.74	2357.14	0.00	0.00	19776.82	31333.58	21423.15	24944.27
84	1428	2-propyl pyrazine	MS/RI/S/O	green，burnt	0.00	0.00	0.00	0.00	0.00	0.00	0.00	0.00	0.00	282.80	0.00	0.00	0.00	0.00	53.40	112.86	0.00	0.00
85	1431	hexyl 2-methylbutyrate	MS/RI/S/O	waxy, unripe fruity, apple and banana	0.00	0.00	0.00	0.00	0.00	193.29	224.03	0.00	204.75	3174.28	136.81	146.13	221.41	2264.82	160.71	382.63	421.01	7336.18
86	1436	ethyl octanoate	MS/RI/S	fruit, fat	207.08	0.00	249.22	204.36	140.31	546.91	946.71	53.53	637.09	0.00	0.00	128.79	0.00	341.88	805.18	608.69	1099.03	0.00
87	1437	(E)-2-octenal	MS/RI/S/O	cucumber fatty green	1034.69	109.19	7139.11	1004.31	301.47	864.69	1228.97	336.59	366.92	0.00	2266.71	2022.38	5099.49	9633.99	1317.76	3621.26	9563.16	11464.11
88	1449	(E)-linalool oxide	MS/RI/S/O	flower	3278.23	2261.74	5553.24	390.06	47.03	18255.55	3841.98	70.12	6086.74	8011.43	2076.28	1346.25	16700.78	16704.47	30678.68	0.00	31774.14	38401.10
89	1450	acetic acid	MS/RI/S/O	sour	0.00	0.00	0.00	68.35	0.00	92.49	113.22	0.00	184.92	1628.18	303.85	0.00	322.25	26.32	864.86	0.00	0.00	0.00
90	1455	furfural	MS/RI/S/O	bread, almond, sweet	2440.95	80.86	972.66	234.03	72.92	0.00	854.28	76.56	200.48	15248.20	993.28	161.11	837.15	11116.69	3518.16	6086.16	1411.90	4167.90
91	1464	cis-3-Hexenyl butyrate	MS/RI/S/O	fresh, green, oily, waxy, apple and pear	0.00	0.00	1679.26	90.07	0.00	7312.63	4061.55	66.75	6174.84	3434.94	111.51	1101.41	1280.08	2520.85	466.98	1001.89	2310.19	16565.22
92	1465	diallyl disulphide	MS/RI/S	green, onion	0.00	0.00	0.00	0.00	0.00	0.00	0.00	0.00	0.00	0.00	0.00	0.00	0.00	0.00	71.22	0.00	134.83	0.00
93	1466	(E)-2-hexen-1-yl butyrate	MS/RI	fruity, apricot, banana	0.00	0.00	0.00	0.00	0.00	1824.50	668.10	0.00	1114.52	1122.56	95.58	104.22	231.08	1130.00	0.00	0.00	378.17	3616.75
94	1467	heptanol	MS/RI/S/O	green	151.80	394.69	1017.15	506.55	106.80	3242.02	1369.91	64.71	2512.73	1532.06	1236.03	376.96	0.00	4982.62	362.93	2499.85	2208.60	4313.06
95	1468	6-methyl-5-hepten-2-ol	MS/RI	sweet, oily, green, coriander	77.57	0.00	0.00	0.00	0.00	899.56	63.15	0.00	64.04	131.64	0.00	0.00	377.29	144.85	0.00	386.74	0.00	1150.07
96	1484	decanal	MS/RI/S	soap, orange	2131.24	1250.01	2093.60	994.47	468.19	1573.59	3065.63	707.75	2160.10	0.00	421.43	1029.82	1466.89	0.00	1756.22	2497.44	3802.26	5161.72
97	1487	2-ethyl-1-hexanol	MS/RI/S/O	sweet fatty fruity	1821.26	146.29	1689.95	1415.14	1431.38	3585.65	4729.88	1849.69	5899.98	1734.04	536.23	11755.33	818.92	2883.99	5368.70	3578.26	4031.51	1176.77
98	1488	α-copaene	MS/RI	wood, spice	0.00	0.00	0.00	0.00	13.79	406.43	160.92	8.88	0.00	0.00	21.49	0.00	0.00	0.00	0.00	222.45	0.00	0.00
99	1490	2-acetylfuran	MS/RI/S/O	nutty and roasted with a sweet	226.97	81.44	220.79	103.87	23.40	70.04	848.32	24.41	365.05	4618.61	231.55	131.70	231.32	6608.62	733.43	465.57	672.35	370.42
100	1493	2-decanone	MS/RI	orange floral fatty peach	167.92	0.00	0.00	87.06	0.00	0.00	0.00	0.00	56.42	2518.65	109.95	0.00	92.87	3169.98	208.89	778.34	598.03	1599.66
101	1495	benzaldehyde	MS/RI/S/O	almond, burnt sugar	5613.92	2097.18	15227.38	4526.08	1336.45	9517.17	10397.69	2364.83	6110.26	36952.14	8415.62	13355.95	8706.01	39766.98	28802.02	38499.87	31233.08	44174.81
102	1497	3,5-diethyl-2-methylpyrazine	MS/RI/S	green, nutty	332.08	0.00	0.00	0.00	0.00	0.00	0.00	0.00	0.00	2248.26	0.00	0.00	0.00	4682.70	0.00	0.00	0.00	0.00
103	1498	pyrrole	MS/RI	sweet, nutty	270.68	0.00	30.09	13.26	0.00	0.00	84.85	31.17	102.53	108.87	114.80	0.00	45.37	195.53	62.32	40.48	0.00	0.00

104	1506	3-nonen-2-one	MS/RI/S/O	spicy fatty woody	76.46	0.00	211.58	95.60	0.00	85.99	58.58	0.00	67.98	3523.74	0.00	136.80	142.82	3380.81	289.96	1233.57	1416.32	4612.08
105	1523	propanoic acid	MS/RI/S	acidic	0.00	30.68	0.00	0.00	0.00	0.00	0.00	0.00	0.00	107.62	0.00	0.00	0.00	163.02	69.97	65.97	71.96	70.67
106	1523	2,6,10,10-tetramethyl-1-oxaspiro[4.5]dec-6-ene	MS/RI	tea，herbal，wet，tobacco，leaf，metallic，woody，spicy	816.20	0.00	0.00	58.48	37.19	58.36	252.22	26.95	26.65	1303.26	98.65	0.00	668.18	624.91	1009.75	311.96	1038.50	2294.56
107	1525	amyl hexanoate	MS/RI	sweet green fruity，pineapple apple pear，fatty	0.00	0.00	0.00	0.00	0.00	37.85	96.32	0.00	36.45	605.72	0.00	0.00	0.00	511.80	0.00	265.20	210.23	1785.10
108	1531	3,5-octadien-2-one	MS/RI/S/O	fruit, fat, mushroom	21.50	0.00	2652.21	0.00	16.90	2239.59	48.31	42.57	13.29	14768.01	0.00	0.00	0.00	14446.46	28.87	14565.56	15133.44	0.00
109	1537	linalool	MS/RI/S/O	flower, lavender	18976.86	14541.94	12483.37	793.68	2405.59	51941.71	18061.88	2537.05	38687.22	20568.46	11181.17	11722.40	46325.43	12784.53	107057.42	76265.55	43993.54	70913.02
110	1542	furfuryl acetate	MS/RI	estery，green banana peel, nasturtium	55.92	0.00	0.00	0.00	0.00	0.00	0.00	0.00	0.00	280.95	0.00	0.00	0.00	1368.18	0.00	0.00	0.00	0.00
111	1544	(E)-2-nonenal	MS/RI/S	soapy, cucumber	0.00	155.81	0.00	0.00	0.00	0.00	0.00	0.00	40.80	0.00	0.00	0.00	654.43	2505.56	0.00	0.00	105.12	0.00
112	1547	(Z)-2-octen-1-ol	MS/RI/S	sweet floral	790.54	0.00	5083.86	0.00	100.69	0.00	3.74	0.00	0.00	0.00	0.00	0.00	0.00	0.00	202.73	1356.16	984.49	0.00
113	1553	1-octanol	MS/RI/S	chemical, metal, burnt	1177.62	993.72	3307.49	195.96	774.07	3883.27	3809.54	596.70	3140.19	7925.03	0.00	0.00	0.00	0.00	1939.95	3020.70	0.00	8440.30
114	1555	(E,E)-2,6-nonadienal	MS/RI/S	cucumber, melon, fatty	141.00	116.33	1733.20	50.50	0.00	0.00	0.00	0.00	0.00	1213.20	207.16	341.34	0.00	0.00	469.24	0.00	0.00	0.00
115	1560	formic acid, octyl ester	MS/RI	orange, citrus	0.00	0.00	0.00	1195.79	0.00	0.00	0.00	0.00	0.00	0.00	1710.44	1909.05	2055.16	8010.10	0.00	979.74	5216.30	603.18
116	1560	5-methylfurfural	MS/RI/S/O	almond, caramel, burnt sugar	1014.64	0.00	223.48	57.96	19.12	51.47	409.85	26.03	110.97	16258.14	432.89	0.00	184.45	13526.65	902.15	2659.76	596.67	0.00
117	1563	(Z)-3-octen-1-ol	MS/RI	fatty，citrus	0.00	0.00	54.35	0.00	0.00	187.40	0.00	0.00	84.58	0.00	0.00	0.00	0.00	0.00	0.00	0.00	0.00	0.00
118	1563	2-methylpropionic acid	MS/RI	rancid, butter, cheese	0.00	0.00	0.00	0.00	0.00	0.00	0.00	0.00	0.00	0.00	0.00	0.00	0.00	0.00	77.59	55.94	0.00	0.00
119	1569	linalyl acetate	MS/RI	sweet, fruit	0.00	0.00	0.00	0.00	0.00	0.00	79.36	0.00	77.07	0.00	0.00	0.00	130.68	0.00	203.50	359.78	299.96	0.00
120	1570	isocaryophyllene	MS/RI	wood	0.00	0.00	57.35	0.00	27.45	0.00	269.52	0.00	9.52	0.00	0.00	0.00	0.00	0.00	0.00	0.00	0.00	0.00
121	1570	(E,E)-3,5-octadien-2-one	MS/RI/S/O	fruity green grassy	247.46	123.91	4779.86	1364.28	131.37	736.56	3374.81	400.98	1579.28	6974.60	4463.29	2927.81	6926.49	6578.76	875.88	7132.75	6117.17	36433.41
122	1575	(E,Z)-2,6-nonadienal	MS/RI/S	cucumber, green	0.00	0.00	972.77	0.00	12.06	0.00	0.00	25.81	13.05	0.00	0.00	0.00	425.91	1961.15	0.00	1756.06	1707.93	3096.69
123	1582	6-methyl-3,5-heptadiene-2-one	MS/RI/S	green, sweet,	0.00	0.00	0.00	0.00	0.00	0.00	0.00	0.00	71.96	6081.52	442.14	295.59	713.47	3768.64	624.69	2146.69	2865.17	3078.27
124	1590	trans-2-decenal	MS/RI	fatty,mushroom	0.00	0.00	620.01	64.91	26.55	232.77	0.00	43.57	37.41	0.00	297.45	494.30	0.00	0.00	0.00	0.00	27.06	0.00

125	1590	β-cyclocitral	MS/RI/S/O	tropical saffron herbal clean rose oxide sweet tobacco damascone fruity	2725.96	835.62	3925.47	1142.64	406.68	2509.72	3934.22	781.57	1318.09	16322.64	3136.30	3554.20	13842.42	14559.69	9245.01	8699.55	11940.99	18584.36
126	1591	terpinen-4-ol	MS/RI	nutmeg, must	753.16	0.00	624.14	36.70	21.27	526.97	98.20	31.52	208.94	1001.59	274.36	406.49	40.20	1168.92	0.00	0.00	0.00	0.00
127	1593	2-acetyl-5-methylfuran	MS/RI	nutty, cocoa, toasted bready	0.00	0.00	0.00	0.00	0.00	29.19	26.87	0.00	0.00	241.26	24.88	0.00	0.00	132.02	54.25	300.81	143.26	569.07
128	1594	β-caryophyllene	MS/RI	wood, spice	0.00	0.00	0.00	69.32	0.00	0.00	0.00	0.00	0.00	0.00	0.00	0.00	0.00	0.00	0.00	0.00	0.00	0.00
129	1595	dimethyl sulfoxide	MS/RI/S	fatty，cheesy，garlic，mushroom	647.70	135.13	295.34	138.76	359.28	367.55	1144.29	571.91	688.76	0.00	416.13	188.79	625.26	0.00	148.15	246.65	0.00	68.11
130	1595	isophorone	MS/RI	sweet, citrus	76.39	186.70	833.92	0.00	0.00	277.70	87.79	5.68	39.88	1850.79	1442.21	76.31	89.54	1566.19	154.22	672.84	809.24	0.00
131	1596	safranal	MS/RI	woody, medicinal, phenolic, spicy and camphoreous with a fruity, herbal nuance	2083.66	85.38	516.51	525.68	314.56	230.51	1861.77	319.43	582.51	10739.91	1094.85	350.38	1772.06	11845.03	7328.46	4870.45	10832.79	6996.02
132	1599	hexyl hexanoate	MS/RI/S/O	herbal fresh cut grass vegetable fruity	0.00	0.00	52.92	0.00	16.17	684.73	817.34	0.00	350.05	12998.32	715.16	636.23	1178.24	7662.20	0.00	0.00	1115.14	14127.32
133	1600	hexadecane	MS/RI	alkane	0.00	0.00	0.00	0.00	0.00	156.96	56.89	0.00	0.00	0.00	0.00	219.56	0.00	1269.16	0.00	0.00	37.61	719.94
134	1600	bornyl acetate	MS/RI	camphoreous, woody, mentholic, berry, soapy	0.00	0.00	48.58	72.30	0.00	27.93	0.00	0.00	0.00	200.74	0.00	51.41	0.00	0.00	0.00	114.07	0.00	0.00
135	1602	octyl butyrate	MS/RI	fresh waxy, fruity,floar	0.00	0.00	0.00	0.00	0.00	0.00	0.00	0.00	0.00	0.00	0.00	0.00	0.00	0.00	0.00	0.00	0.00	0.00
136	1606	(3Z)-3-hexenyl (2E)-2-butenoate	MS/RI	green, vegetable	0.00	0.00	0.00	0.00	0.00	0.00	35.22	0.00	0.00	0.00	0.00	0.00	0.00	136.90	52.78	0.00	0.00	0.00
137	1610	(E)-2-octenol	MS/RI/S	citrus,fatty	0.00	182.36	26.90	287.74	43.37	723.49	2197.86	157.09	2394.25	1111.46	199.17	377.00	358.89	1535.61	0.00	0.00	0.00	1456.04
138	1615	(E,E)-2,4-octadienal	MS/RI	green，fruity，melon，citrus，fatty，tallow	0.00	0.00	549.86	41.60	0.00	0.00	71.87	0.00	31.11	1040.53	8.53	146.89	0.00	1257.61	163.29	0.00	0.00	1168.21
139	1616	1-ethyl-1H-pyrrole-2-carboxaldehyde	MS/RI/S/O	burnt	19832.33	177.93	0.00	0.00	764.47	126.37	7039.34	0.00	2688.52	39728.28	1140.96	82.30	3023.65	0.00	0.00	15317.71	4429.64	4176.47
140	1616	β-terpineol	MS/RI	pungent earthy woody	0.00	0.00	0.00	0.00	0.00	0.00	0.00	0.00	0.00	0.00	0.00	0.00	0.00	0.00	70.03	0.00	0.00	0.00

141	1616	5H-5-methyl-6,7-dihydrocyclopentapyrazine	MS/RI	musty, nutty, roasted and grainy with coffee notes	0.00	0.00	0.00	0.00	0.00	0.00	20.83	0.00	0.00	400.49	0.00	0.00	0.00	499.47	0.00	0.00	0.00	0.00
142	1619	carbitol	MS/RI	slightly ethereal	146.45	0.00	57.88	11.39	0.00	29.39	0.00	17.02	30.36	144.01	78.10	139.83	0.00	0.00	40.23	0.00	0.00	128.48
143	1619	butanoic acid	MS/RI/S	rancid, cheese, sweat	0.00	0.00	0.00	22.17	0.00	0.00	0.00	0.00	0.00	0.00	0.00	0.00	0.00	0.00	0.00	0.00	0.00	0.00
144	1620	p-menth-1-en-9-al	MS/RI	spicy，herbal	203.79	0.00	0.00	0.00	0.00	136.71	116.98	0.00	16.17	233.18	200.88	0.00	0.00	1978.52	832.09	562.13	198.99	418.01
145	1622	2-methyl-benzaldehyde	MS/RI	cherry	173.91	39.21	443.54	119.56	19.54	46.06	488.54	76.70	72.15	715.91	0.00	0.00	38.47	3639.54	206.31	0.00	27.61	0.00
146	1622	2-acetyl pyrazine	MS/RI	popcorn, chocolate, hazelnut, coffee	0.00	0.00	0.00	0.00	0.00	0.00	0.00	0.00	0.00	160.46	0.00	0.00	0.00	405.49	0.00	0.00	0.00	0.00
147	1622	linalyl isobutyrate	MS/RI	floral, fruity, sweet, berry, citrus	0.00	0.00	0.00	39.24	0.00	0.00	0.00	0.00	0.00	0.00	0.00	0.00	0.00	0.00	0.00	0.00	0.00	0.00
148	1625	phenylethanal	MS/RI/S/O	honey, sweet	380.10	714.69	2100.38	382.04	373.65	1104.85	2591.04	1013.84	1428.06	9561.37	11646.94	60941.27	8762.51	10684.54	26447.33	41634.39	29539.64	38919.09
149	1626	1-methyl-1H-pyrrole-2-carboxaldehyde	MS/RI	roasted nutty	17.01	0.00	0.00	127.21	90.20	23.34	922.11	27.81	0.00	949.00	0.00	65.13	0.00	2393.23	142.15	0.00	0.00	208.70
150	1627	3-methyl-benzaldehyde	MS/RI	fruity, nutty	0.00	0.00	0.00	35.89	0.00	55.21	287.60	42.53	0.00	0.00	349.53	246.97	0.00	609.25	630.90	0.00	1091.45	243.43
151	1631	menthol	MS/RI	minty	0.00	0.00	0.00	0.00	0.00	0.00	0.00	0.00	0.00	0.00	0.00	0.00	0.00	0.00	0.00	0.00	226.83	0.00
152	1636	2-hydroxybenzaldehyde	MS/RI	spicy, medicinal and astringent	0.00	0.00	680.83	0.00	0.00	107.55	0.00	0.00	0.00	675.95	0.00	0.00	0.00	633.48	0.00	0.00	1513.05	0.00
153	1645	acetophenone	MS/RI/S	bitter almond	0.00	155.12	2647.52	522.59	355.54	1757.49	2479.69	640.72	2588.18	7149.44	0.00	0.00	0.00	8305.25	6532.14	0.00	44.26	0.00
154	1646	alloaromadendrene	MS/RI	woody	0.00	0.00	0.00	0.00	0.00	40.17	0.00	0.00	0.00	0.00	0.00	36.67	0.00	0.00	0.00	0.00	0.00	0.00
155	1646	3-hexenyl hexanoate	MS/RI/S/O	green, fruity, fatty, tropical and pulpy with citrus	243.73	69.36	0.00	99.80	1430.91	8618.12	13040.56	217.57	8808.01	21212.07	1391.28	4356.86	5486.03	7501.28	1004.80	0.00	6166.10	33066.93
156	1646	α-caryophyllene	MS/RI/S	woody	0.00	0.00	0.00	0.00	0.00	89.96	52.37	0.00	32.24	0.00	0.00	0.00	0.00	0.00	0.00	0.00	31.36	32.88
157	1647	γ-butyrolactone	MS/RI	caramel, fatty	297.13	0.00	232.24	54.45	27.43	66.90	250.04	173.92	130.34	0.00	152.75	125.70	0.00	266.02	279.38	0.00	219.21	180.22
158	1648	dehydrolinalool	MS/RI/S/O	mouldy	12891.50	0.00	2698.66	126.84	107.79	3209.27	5237.56	965.88	2189.70	14954.26	27434.87	3419.78	11075.60	3983.12	8733.97	31301.23	10376.20	28837.41
159	1653	para-tolualdehyde	MS/RI	fruity，cherry	0.00	0.00	0.00	0.00	36.31	0.00	130.89	19.04	348.68	3188.42	0.00	0.00	0.00	0.00	0.00	0.00	222.00	1702.60
160	1655	4-allylanisole	MS/RI	licorice, anise	93.92	0.00	200.39	45.61	61.55	168.91	192.38	0.00	146.53	0.00	0.00	109.28	42.05	0.00	249.99	156.74	391.23	319.61
161	1657	2,6-octadienoic acid, 3,7-dimethyl-, methyl ester, (Z)-	MS/RI	floral, herbal, citrus, fruity, green,	0.00	0.00	0.00	0.00	0.00	0.00	0.00	0.00	0.00	70.91	0.00	0.00	0.00	77.64	0.00	0.00	0.00	0.00

				geranium																		
162	1658	β-farnesene	MS/RI/S	wood, citrus, sweet	48.04	0.00	0.00	0.00	0.00	154.46	0.00	0.00	210.71	7482.46	3022.32	17098.88	0.00	1132.79	0.00	0.00	0.00	0.00
163	1659	furfuryl alcohol	MS/RI/S	burnt	286.23	0.00	0.00	105.72	15.56	0.00	184.85	0.00	84.84	0.00	0.00	0.00	0.00	573.10	626.41	275.99	167.67	193.67
164	1659	2-butyl-2-octenal	MS/RI/S	green vegetable, leafy cucumber, oily, herbal, fatty, watercress, violet	0.00	0.00	0.00	0.00	0.00	0.00	253.70	0.00	0.00	514.68	55.51	0.00	0.00	0.00	429.15	2200.58	765.94	312.38
165	1660	cis-beta-farnesene	MS/RI	citrus，green	0.00	0.00	148.86	0.00	44.99	248.94	64.25	0.00	310.36	0.00	0.00	0.00	1419.67	0.00	478.63	1184.99	692.23	7288.90
166	1660	6,10-dimethyl-2-undecanone	MS/RI	citronella，soapy	0.00	0.00	42.59	0.00	0.00	0.00	157.86	0.00	0.00	1571.07	0.00	54.64	89.15	775.12	157.87	311.14	640.59	1160.83
167	1660	(E)-2-hexen-1-yl hexanoate	MS/RI	apple，pear，kiwi，leafy	103.17	0.00	0.00	0.00	44.54	0.00	1481.95	0.00	1164.70	8566.10	550.08	235.75	477.70	5612.40	387.50	0.00	1421.37	12284.13
168	1664	(Z)-3-nonen-1-ol	MS/RI/S	green, fat	0.00	130.46	600.53	0.00	27.84	932.18	367.11	0.00	707.81	0.00	0.00	0.00	1459.87	0.00	1005.65	3482.83	289.03	5318.51
169	1665	3-methylbutanoic acid	MS/RI/S	sweat, acid, rancid	0.00	0.00	0.00	0.00	0.00	0.00	0.00	0.00	0.00	180.41	0.00	0.00	0.00	0.00	1260.27	0.00	542.62	0.00
170	1666	L-trans-pinocarveol	MS/RI	woody, balsamic, fennel	0.00	0.00	0.00	0.00	0.00	105.65	0.00	0.00	0.00	0.00	0.00	0.00	0.00	0.00	224.58	0.00	1933.87	212.76
171	1667	(Z)-3,7-dimethylocta-2,6-dienal	MS/RI	lemon	0.00	0.00	483.69	84.19	0.00	444.98	115.56	0.00	311.15	75.37	89.20	174.38	69.98	0.00	1386.44	6133.18	0.00	11609.30
172	1668	nonanol	MS/RI/S	fat, green	0.00	477.91	1144.70	151.76	0.00	2811.67	897.37	0.00	1838.16	2251.79	0.00	0.00	0.00	0.00	0.00	0.00	0.00	9422.97
173	1673	benzyl formate	MS/RI	fresh cherry, with a berry strawberry fruity nuance	0.00	0.00	395.02	32.16	0.00	0.00	0.00	0.00	0.00	1116.05	0.00	0.00	0.00	867.69	0.00	0.00	0.00	0.00
174	1677	ketoisophorone	MS/RI	citrus, floral, musty, tea like with green sweet fruity nuances	0.00	0.00	139.07	77.14	0.00	62.37	128.72	9.73	61.47	0.00	238.35	86.91	0.00	0.00	0.00	0.00	865.80	0.00
175	1677	trans-geranic acid methyl ester	MS/RI	waxy green fruity flower	0.00	0.00	28.83	0.00	0.00	757.61	0.00	0.00	550.96	958.57	0.00	0.00	66.14	781.50	0.00	8399.15	1505.73	2487.64
176	1681	geranyl formate	MS/RI/S	green, floral, citrus, waxy, fruity, apple and apricot-like	0.00	0.00	0.00	0.00	76.98	15.01	0.00	0.00	0.00	0.00	0.00	0.00	0.00	0.00	0.00	670.62	548.61	4252.68
177	1683	5-ethenyldihydro-5-methyl-2(3H)-furanone	MS/RI	fruity, minty	0.00	0.00	155.79	0.00	0.00	0.00	0.00	0.00	0.00	669.67	0.00	0.00	0.00	618.08	0.00	0.00	0.00	0.00

178	1686	(E,E)-2,4-nonadienal	MS/RI/S	fat, green	0.00	0.00	71.33	0.00	0.00	0.00	0.00	0.00	0.00	0.00	0.00	0.00	0.00	1173.62	87.88	0.00	1203.08	55.20
179	1688	α-terpineol	MS/RI	pine, terpene, lilac, citrus, woody, floral	3302.55	0.00	1899.88	62.46	0.00	575.39	1428.77	173.60	0.00	0.00	0.00	0.00	4242.24	2850.85	11439.82	5239.07	0.00	4745.69
180	1694	γ-caprolactone	MS/RI	sweet, creamy, tobacco	0.00	0.00	0.00	6.95	0.00	48.26	260.55	0.00	81.02	110.78	667.95	959.46	381.00	1229.64	128.10	216.09	370.41	1218.36
181	1696	propiophenone	MS/RI	fruity	0.00	0.00	0.00	13.07	0.00	0.00	0.00	0.00	0.00	4927.79	204.14	163.61	0.00	5195.47	261.84	542.27	246.96	707.30
182	1706	(Z)-3-hexen-1-yl (E)-2-hexenoate	MS/RI	fruity, herbal, artichoke, tea	0.00	0.00	0.00	0.00	0.00	57.01	86.41	0.00	0.00	538.43	0.00	0.00	0.00	0.00	0.00	0.00	157.82	805.36
183	1710	(E,E)-2,4-decadienal	MS/RI	fat,fried and potato	0.00	0.00	310.22	0.00	0.00	0.00	0.00	0.00	0.00	821.95	160.59	71.12	0.00	685.83	0.00	364.92	1019.42	0.00
184	1710	2,4-dimethylbenzaldehyde	MS/RI	naphthyl, cherry, almond, spice and vanilla	0.00	0.00	0.00	20.02	0.00	14.98	0.00	0.00	37.32	3201.59	273.80	0.00	0.00	165.28	1083.58	0.00	2071.61	3399.89
185	1714	benzyl acetate	MS/RI	sweet, floral, fruity, jasmin, fresh	0.00	0.00	19.46	98.48	0.00	142.59	72.55	0.00	35.94	4045.27	0.00	88.22	0.00	2848.07	1112.95	848.46	1935.49	1460.76
186	1714	β-bisabolene	MS/RI	balsamic	0.00	0.00	0.00	0.00	0.00	0.00	0.00	0.00	23.64	2141.62	572.98	3259.32	260.26	0.00	52.66	69.35	69.59	1644.32
187	1715	(Z)-3-hexen-1-yl (Z)-3-hexenoate	MS/RI/S/O	green, tomato, leaf, pear, melon, metallic, fennel, tropical	57.32	0.00	114.50	0.00	200.07	1614.91	1021.48	0.00	631.38	2334.09	103.92	130.27	210.63	622.47	0.00	220.30	376.82	2387.57
188	1715	(E)-citral	MS/RI/S	lemon, mint	87.09	0.00	1337.52	161.00	0.00	1634.31	324.36	0.00	1593.09	2755.44	0.00	0.00	371.81	1637.07	2829.03	7687.02	4450.33	18420.46
189	1717	citral	MS/RI/S	lemon	0.00	462.55	0.00	0.00	18.91	25.30	17.57	11.74	24.94	0.00	189.01	318.50	0.00	0.00	43.63	1628.43	0.00	0.00
190	1718	carvone	MS/RI/S	minty	0.00	0.00	0.00	0.00	0.00	308.12	0.00	0.00	0.00	0.00	0.00	237.07	0.00	0.00	73.46	0.00	69.37	0.00
191	1718	naphthalene	MS/RI	tar	596.90	97.13	566.20	332.95	127.84	695.59	955.53	0.00	384.11	0.00	218.72	520.39	385.75	2442.52	1530.70	1346.65	2401.58	0.00
192	1720	epoxylinalol	MS/RI	floral, honey	0.00	0.00	0.00	5.57	12.73	8.50	0.00	0.00	0.00	0.00	0.00	0.00	0.00	0.00	187.01	0.00	111.68	0.00
193	1722	dodecanal	MS/RI	soapy, fat, citrus	0.00	0.00	100.01	0.00	29.33	0.00	0.00	0.00	0.00	439.78	0.00	20.87	0.00	200.27	118.49	202.74	456.15	915.91
194	1722	2-thiophenecarboxaldehyde	MS/RI/S	sulfurous	0.00	0.00	0.00	0.00	0.00	0.00	110.08	26.03	0.00	0.00	0.00	0.00	0.00	577.05	0.00	552.42	0.00	0.00
195	1730	δ-cadinene	MS/RI	thyme herbal woody dry	113.33	54.46	54.77	20.21	289.28	3052.98	1396.48	201.83	451.07	220.40	163.43	0.00	199.22	0.00	244.88	10083.47	569.88	1853.40

196	1731	3,6-(E,Z)-Nonadien-1-ol	MS/RI	fresh green, waxy, melon, with fruity pear notes	0.00	0.00	0.00	0.00	0.00	119.53	0.00	0.00	0.00	0.00	25.95	0.00	194.78	0.00	161.34	1452.22	226.64	999.39
197	1741	(E)-linalool oxide (pyranoid)	MS/RI/S/O	woody	487.67	438.78	369.19	67.27	202.44	4789.10	1671.02	200.73	2457.91	4002.92	2547.11	1095.53	3230.61	1838.38	10543.09	17411.31	11017.36	21123.30
198	1745	2(5H)-furanone	MS/RI/S	buttery	62.23	0.00	0.00	0.00	0.00	0.00	0.00	0.00	0.00	0.00	0.00	0.00	0.00	0.00	130.30	0.00	0.00	0.00
199	1745	methyl salicylate	MS/RI/S/O	peppermint	1479.34	1144.03	4935.90	175.61	58.60	12756.85	4690.50	216.91	5352.75	15710.86	1276.76	3378.56	8452.28	11085.14	30663.37	28228.38	18741.17	54968.35
200	1748	α-farnesene	MS/RI	citrus herbal	0.00	0.00	55.29	0.00	0.00	33.51	0.00	0.00	67.27	2760.69	902.77	7090.74	9635.10	0.00	171.30	226.37	0.00	3134.49
201	1749	methyl 2-phenylacetate	MS/RI	floral, honey, spice, waxy and sweet	0.00	0.00	0.00	0.00	0.00	16.27	45.18	11.08	22.27	2569.46	99.34	39.48	0.00	3514.35	140.84	368.29	201.69	0.00
202	1754	citronellol	MS/RI	floral, citrus	0.00	0.00	0.00	30.14	0.00	0.00	0.00	0.00	0.00	0.00	0.00	0.00	0.00	0.00	520.77	794.55	0.00	16.24
203	1755	2-undecenal	MS/RI	citrus , soapy	0.00	0.00	0.00	0.00	104.80	0.00	0.00	0.00	0.00	424.07	99.06	0.00	144.67	0.00	0.00	272.43	539.04	1212.84
204	1770	nerol	MS/RI/S/O	sweet	1575.56	418.41	912.23	0.00	123.65	2246.50	229.12	105.53	1079.58	1542.86	729.55	1318.02	1000.20	5591.35	3293.39	7650.89	3479.02	123818.97
205	1770	γ-heptalactone, 4-heptanolide	MS/RI	nut, fat, fruit	0.00	0.00	0.00	0.00	0.00	0.00	17.23	0.00	4.15	0.00	0.00	0.00	0.00	218.42	0.00	0.00	0.00	0.00
206	1771	benzyl isobutanoate	MS/RI	fruity, sweet, ripe berry	0.00	0.00	0.00	0.00	0.00	0.00	0.00	0.00	0.00	0.00	0.00	0.00	0.00	0.00	0.00	0.00	0.00	192.48
207	1773	α-curcumene	MS/RI	herb	44.34	0.00	253.68	0.00	13.26	193.56	150.18	0.00	182.12	1846.54	201.38	1088.35	288.65	919.93	318.00	401.74	787.01	708.72
208	1775	phenethyl formate	MS/RI	sweet, green, watercress, unripe banana, narcissus, phenolic	0.00	0.00	0.00	36.84	0.00	31.66	0.00	13.47	0.00	0.00	0.00	0.00	0.00	0.00	323.36	0.00	441.19	0.00
209	1776	2-phenyl-2-propanol	MS/RI	sweet	82.75	0.00	0.00	36.14	0.00	1.13	23.16	0.00	133.74	0.00	0.00	0.00	0.00	0.00	0.00	0.00	0.00	0.00
210	1778	α-methylphenethyl alcohol	MS/RI	sweet, pineapple	0.00	0.00	0.00	0.00	0.00	0.00	0.00	0.00	0.00	0.00	0.00	0.00	0.00	0.00	0.00	28.60	0.00	0.00
211	1782	β-sesquiphellandrene	MS/RI	wood	269.70	198.31	0.00	0.00	0.00	0.00	56.29	0.00	304.25	352.32	61.15	494.03	0.00	0.00	0.00	0.00	0.00	206.93
212	1784	(Z)-4-decen-1-ol	MS/RI	waxy, fatty, fruity	0.00	0.00	0.00	0.00	0.00	0.00	0.00	0.00	139.20	0.00	0.00	0.00	0.00	0.00	0.00	0.00	0.00	0.00
213	1791	phenylethyl acetate	MS/RI	floral, rose, sweet, honey, fruity , tropical	144.73	0.00	0.00	21.70	8.77	93.13	0.00	0.00	43.28	4781.14	300.06	430.42	0.00	2693.33	833.26	1364.29	931.33	2430.18
214	1794	4-acetyltoluene	MS/RI	sweet, creamy, fruity, cherry	0.00	0.00	0.00	0.00	0.00	0.00	0.00	0.00	0.00	0.00	0.00	0.00	0.00	0.00	68.16	0.00	0.00	506.03
215	1797	2,4-decadienal	MS/RI	orange, sweet, fresh, citrus, fatty	0.00	0.00	113.20	0.00	0.00	0.00	0.00	0.00	0.00	0.00	0.00	121.76	126.06	709.28	150.70	298.69	418.08	2074.75

216	1803	hexyl octanoate	MS/RI	green, apple, fruity, berry，fresh	0.00	0.00	0.00	0.00	0.00	0.00	0.00	0.00	0.00	0.00	0.00	0.00	0.00	0.00	0.00	0.00	0.00	186.53
217	1804	methyl laurate	MS/RI	fatty coconut	0.00	0.00	0.00	0.00	0.00	0.00	0.00	0.00	10.51	0.00	21.08	0.00	0.00	0.00	0.00	0.00	0.00	187.34
218	1807	(E)-2-dodecenal	MS/RI	fatty, sweet	0.00	0.00	141.50	0.00	0.00	0.00	0.00	11.44	0.00	0.00	0.00	0.00	0.00	0.00	0.00	0.00	0.00	0.00
219	1811	(E)-geranyl acetone	MS/RI	floral fruity	3736.11	0.00	0.00	0.00	306.80	1956.69	0.00	0.00	0.00	0.00	872.26	0.00	0.00	0.00	0.00	0.00	0.00	0.00
220	1814	2-tridecanone	MS/RI	fatty, earthy, dairy, ketonic, cheesy	55.47	0.00	56.62	0.00	0.00	0.00	0.00	0.00	0.00	0.00	0.00	0.00	0.00	73.28	42.89	50.36	57.61	82.78
221	1814	(E)-beta-damascenone	MS/RI/S/O	apple rose honey tobacco sweet	311.89	0.00	0.00	0.00	0.00	0.00	47.17	13.10	0.00	1571.69	132.83	17.83	0.00	448.10	5627.31	2067.99	1427.41	1711.68
222	1815	anethole	MS/RI	sweet, anise, licorice, medicinal	0.00	0.00	371.56	27.16	33.43	0.00	326.25	61.57	228.62	0.00	0.00	86.22	0.00	0.00	0.00	223.27	0.00	0.00
223	1815	nerylacetone	MS/RI	fatty, metallic	0.00	0.00	9653.70	1123.30	116.88	355.44	1774.30	211.78	1440.93	0.00	0.00	2967.31	2335.77	0.00	5729.71	9621.92	0.00	18744.57
224	1817	cis-anethole	MS/RI	sweet anise licorice medicinal	0.00	0.00	0.00	0.00	0.00	0.00	0.00	0.00	0.00	0.00	65.16	0.00	0.00	0.00	0.00	0.00	0.00	0.00
225	1820	α-methylbenzyl alcohol	MS/RI	chemical, medicinal, with a balsamic vanilla woody nuance	0.00	0.00	0.00	19.85	0.00	34.57	0.00	0.00	0.00	67.97	0.00	0.00	0.00	0.00	83.83	0.00	260.78	0.00
226	1822	undecanol	MS/RI	soapy waxy sweet	0.00	0.00	0.00	0.00	0.00	0.00	0.00	0.00	0.00	0.00	171.74	0.00	0.00	0.00	0.00	0.00	764.64	0.00
227	1823	dec-2-en-1-ol	MS/RI	waxy, fresh air, citrus, rose rue	286.06	0.00	0.00	63.34	0.00	0.00	0.00	0.00	25.06	0.00	0.00	0.00	0.00	0.00	0.00	0.00	10.37	0.00
228	1824	beta-damascone	MS/RI/S/O	fruity，floral，black currant，plum，rose，honey，tobacco	0.00	0.00	269.16	0.00	0.00	0.00	18.18	0.00	0.00	565.43	21.84	35.46	58.11	171.80	19.98	121.41	207.36	122.31
229	1824	tridecanal	MS/RI	flower, sweet	0.00	0.00	0.00	0.00	0.00	0.00	0.00	0.00	0.00	0.00	57.95	0.00	0.00	0.00	0.00	0.00	0.00	0.00
230	1827	6,6-dimethyl-2-norpinene-2-ethanol	MS/RI	sweet, balsamic, citrus, pine, herbal	0.00	0.00	0.00	0.00	0.00	0.00	0.00	0.00	0.00	81.37	0.00	0.00	0.00	0.00	0.00	0.00	0.00	0.00
231	1829	hexanoic acid	MS/RI/S/O	sour fatty	94.20	0.00	158.99	0.00	0.00	282.41	240.93	0.00	77.76	6272.49	424.44	576.23	0.00	5150.91	2003.24	5589.32	5636.27	0.00
232	1832	phenethyl pivalate	MS/RI	balsamic, tea, rose, spicy, geranium, green grassy, waxy	0.00	0.00	0.00	0.00	0.00	0.00	0.00	0.00	0.00	52.45	0.00	0.00	0.00	0.00	0.00	0.00	0.00	0.00
233	1833	1-furfuryl pyrrole	MS/RI/S/O	vegetative, onion, sharp	464.52	0.00	27.34	0.00	0.00	9.09	93.79	0.00	66.09	3118.10	29.16	17.59	0.00	2235.60	51.08	262.77	21.85	54.27

				and metallic																		
234	1838	(Z)-3-hexen-1-yl octanoate	MS/RI	green, fruity, winey, oily, asparagus, artichoke, metallic	0.00	0.00	0.00	0.00	22.62	74.56	56.57	0.00	114.84	0.00	0.00	65.37	0.00	80.80	0.00	0.00	0.00	556.23
235	1841	4-(2,6,6-Trimethylcyclohexa-1,3-dienyl)butan-2-one	MS/RI	floral	0.00	0.00	0.00	0.00	0.00	0.00	0.00	0.00	0.00	0.00	14.64	0.00	0.00	0.00	0.00	0.00	0.00	0.00
236	1842	ionone	MS/RI	violet sweet floral woody	36.14	0.00	0.00	0.00	0.00	0.00	0.00	0.00	0.00	0.00	0.00	0.00	0.00	6773.72	0.00	0.00	0.00	0.00
237	1846	carveol	MS/RI	minty, green weedy, herbal, plastic, spicy, earthy, mahogany	0.00	0.00	0.00	0.00	0.00	85.85	0.00	0.00	0.00	0.00	0.00	129.99	83.22	140.05	92.46	148.80	79.10	0.00
238	1850	2-hexanoylfuran	MS/RI	sweet, fruity, green, waxy, beany	0.00	0.00	0.00	0.00	0.00	0.00	15.21	0.00	0.00	0.00	0.00	0.00	0.00	0.00	0.00	0.00	0.00	0.00
239	1851	2-furanacrolein	MS/RI	green grassy, fruity, spicy, vanilla, nutty, woody, cinnamon	0.00	0.00	0.00	0.00	0.00	0.00	0.00	0.00	0.00	262.52	0.00	0.00	0.00	297.30	0.00	0.00	0.00	0.00
240	1853	trans-2-Hexenyl n-octanoate	MS/RI	pear	0.00	0.00	0.00	0.00	0.00	0.00	0.00	0.00	0.00	0.00	0.00	0.00	0.00	20.42	0.00	0.00	0.00	0.00
241	1854	dihydro-β-Ionone	MS/RI	FLORAL,woody, seedy, berry raspberry, with leafy, spicy nuances	90.49	0.00	0.00	8.49	0.00	0.00	0.00	0.00	19.86	0.00	25.07	0.00	0.00	291.79	325.88	460.06	438.54	0.00
242	1854	dihydro-α-ionone	MS/RI	creamy, berry, floral, woody, orris, earthy, violet	0.00	0.00	0.00	0.00	0.00	0.00	0.00	0.00	0.00	0.00	0.00	0.00	0.00	0.00	65.02	0.00	0.00	0.00
243	1855	1-methyl-naphthalene	MS/RI	chemical	0.00	0.00	0.00	45.73	34.80	0.00	202.07	57.90	155.44	0.00	0.00	0.00	54.82	0.00	0.00	0.00	0.00	0.00
244	1857	methyl β-phenylpropionate	MS/RI	honey, fruity, wine, balsam, floral	0.00	0.00	0.00	0.00	0.00	0.00	0.00	0.00	0.00	0.00	0.00	0.00	0.00	379.95	0.00	0.00	0.00	0.00
245	1860	geraniol	MS/RI/S/O	floral, rosy, waxy and perfumey with a fruity, peach-like nuance	0.00	11419.54	1682.13	1458.19	0.00	33944.61	6681.58	0.00	44227.66	13807.54	0.00	0.00	10166.37	0.00	29200.31	55092.42	35166.46	0.00

246	1861	2′, 4′-dimethylacetophenone	MS/RI	floral, woody, sweet, mimosa, minty	0.00	0.00	41.75	0.00	46.45	25.61	9.07	79.30	0.00	1170.70	31.49	0.00	0.00	58.47	35.13	0.00	0.00	0.00
247	1862	o-guaiacol	MS/RI	phenolic, smoke, spice, vanilla, woody	0.00	0.00	0.00	38.90	0.00	0.00	0.00	0.00	0.00	27.03	0.00	0.00	0.00	0.00	0.00	23.74	40.08	0.00
248	1863	α-Ionone	MS/RI/S/O	sweet, woody, floral, violet, orris, tropical fruity	0.00	448.06	10464.85	0.00	0.00	6.63	1280.73	0.00	1117.79	0.00	863.36	0.00	0.00	12411.77	0.00	284.97	0.00	0.00
249	1865	benzyl alcohol	MS/RI/S/O	sweet, flower	295.32	1763.20	1752.96	1318.86	178.61	6415.53	1801.29	92.62	1387.87	5020.91	980.33	1411.99	2080.08	3999.10	8811.32	11995.50	16682.58	20256.25
250	1867	p-acetylethylbenzene	MS/RI	floral, hawthorn	0.00	0.00	0.00	0.00	0.00	12.03	0.00	0.00	50.10	0.00	0.00	99.58	0.00	0.00	0.00	0.00	0.00	0.00
251	1877	2-methyl-naphthalene	MS/RI	sweet floral	0.00	0.00	0.00	0.00	71.36	0.00	0.00	58.94	47.11	0.00	122.54	0.00	122.98	0.00	0.00	0.00	0.00	0.00
252	1877	Propanoic acid, 2-methyl-, 2-phenylethyl ester	MS/RI	honey, floral, fruity, tea, rose,peach,pastry	0.00	0.00	0.00	0.00	0.00	0.00	0.00	0.00	0.00	1560.69	676.79	615.84	56.71	593.87	0.00	0.00	0.00	0.00
253	1879	Furfural acetone	MS/RI	sweet, nutty, powdery, vanilla, coumarin, creamy	0.00	0.00	0.00	0.00	0.00	0.00	0.00	0.00	0.00	53.58	0.00	0.00	0.00	0.00	0.00	0.00	0.00	0.00
254	1881	γ-octalactone	MS/RI	coconut, fatty	0.00	0.00	0.00	0.00	0.00	0.00	38.77	0.00	0.00	1021.12	114.07	140.38	0.00	415.47	0.00	118.96	180.67	590.01
255	1885	dihydropseudoionone	MS/RI	floral, fruity, tropical, green pear apple banana citrus	0.00	0.00	0.00	0.00	0.00	0.00	0.00	0.00	0.00	19742.78	0.00	0.00	0.00	0.00	0.00	0.00	0.00	91.77
256	1894	butanoic acid, 3-methyl-, phenylmethyl ester	MS/RI	sweet, fruity, apple, pineapple, herbal	0.00	0.00	0.00	0.00	0.00	0.00	0.00	0.00	0.00	518.66	0.00	38.98	0.00	0.00	0.00	0.00	277.10	0.00
257	1895	dimethyl sulfone	MS/RI	sulfur, burnt	0.00	0.00	0.00	0.00	0.00	0.00	0.00	0.00	0.00	0.00	0.00	0.00	0.00	76.74	10.44	0.00	0.00	0.00
258	1895	geranyl isovalerate	MS/RI	fruit, rose, apple	0.00	0.00	0.00	0.00	0.00	0.00	0.00	0.00	0.00	0.00	0.00	0.00	0.00	0.00	0.00	274.64	0.00	0.00
259	1899	trans-2-undecen-1-ol	MS/RI	floral, rose, waxy, fresh fruity	78.09	0.00	264.60	0.00	0.00	65.86	0.00	0.00	174.06	0.00	0.00	0.00	0.00	0.00	0.00	0.00	213.85	510.58
260	1907	benzeneacetaldehyde, α-ethylidene	MS/RI	green, vegetative, floral, cocoa and nutty	122.24	0.00	0.00	0.00	0.00	9.82	64.85	0.00	0.00	1715.76	91.83	105.96	0.00	1048.19	3328.15	3443.03	1932.26	3774.36
261	1910	2,6-di-tert-butyl-p-methylphenol	MS/RI	mild phenolic camphor	198.31	97.79	202.73	0.00	0.00	45.21	54.64	0.00	54.69	294.78	64.83	55.04	98.46	194.06	207.24	228.36	236.30	352.96

262	1912	2-phenylethanol	MS/RI/S/O	floral, sweet, rosey and bready	0.00	1387.55	784.84	1007.22	136.76	7477.69	1698.71	181.62	1641.94	11422.80	6694.73	16387.96	2507.01	3231.53	14786.78	33307.05	21157.21	30291.35
263	1914	cubebol	MS/RI	spicy, minty	0.00	0.00	0.00	0.00	0.00	54.58	38.42	0.00	4.33	85.56	0.00	9.47	0.00	0.00	88.17	0.00	64.79	45.59
264	1916	α-calacorene	MS/RI	woody	0.00	0.00	77.27	0.00	0.00	35.40	85.38	0.00	26.79	0.00	0.00	0.00	0.00	0.00	221.82	1996.72	262.91	773.52
265	1917	β-Lonone	MS/RI/S/O	woody, floral, berry, fruity	4498.02	1596.90	7952.29	1145.08	230.55	2057.47	5342.28	883.39	1526.95	22400.27	3934.08	4941.61	4177.54	14914.45	11652.62	14028.20	20241.21	28705.46
266	1920	benzeneacetaldehyde, α-(2-methylpropylidene)-	MS/RI	sweet, cocoa, nutty, rose, powdery	52.48	0.00	0.00	0.00	0.00	0.00	0.00	0.00	0.00	686.22	0.00	0.00	0.00	368.47	2205.45	2329.52	1119.41	1107.10
267	1927	calamenene	MS/RI	herb, spice	0.00	32.82	0.00	0.00	147.51	1334.44	968.78	82.15	116.30	655.23	0.00	52.22	63.95	59.22	0.00	0.00	0.00	1246.34
268	1928	isoamyl benzoate	MS/RI	sweet, fruity with a green tropical nuance	0.00	0.00	0.00	0.00	0.00	0.00	0.00	0.00	0.00	209.05	0.00	13.90	0.00	101.22	0.00	0.00	0.00	81.12
269	1936	3-Phenyl-1-propanol, acetate	MS/RI	balsamic, floral, fruity, sappy, spicy and cinnamic	0.00	0.00	0.00	62.10	0.00	0.00	0.00	31.12	0.00	0.00	0.00	0.00	0.00	0.00	0.00	0.00	0.00	0.00
270	1940	Piperitenone oxide	MS/RI	herbal, minty	0.00	0.00	0.00	0.00	0.00	0.00	159.28	0.00	0.00	0.00	0.00	0.00	0.00	0.00	0.00	0.00	72.97	0.00
271	1945	cis-3-hexenoic acid	MS/RI	green grass, sweaty, fruity, cheesy acid	0.00	0.00	0.00	0.00	0.00	0.00	0.00	0.00	0.00	356.32	0.00	0.00	0.00	0.00	0.00	0.00	0.00	102.87
272	1955	heptanoic acid	MS/RI/S	cheesy, fruity, fatty	0.00	0.00	35.81	0.00	0.00	0.00	25.82	0.00	0.00	689.41	0.00	32.00	0.00	687.32	278.57	267.92	722.42	831.69
273	1956	creosol	MS/RI	vaniila, spice, woody	0.00	0.00	0.00	2.45	0.00	0.00	0.00	0.00	0.00	0.00	0.00	0.00	0.00	0.00	0.00	0.00	0.00	0.00
274	1956	benzothiazole	MS/RI	meaty, beefy and coffee-like	0.00	0.00	0.00	14.11	40.32	85.67	70.34	48.39	139.53	505.16	0.00	96.23	0.00	418.41	252.86	350.09	0.00	345.38
275	1958	2-phenylethyl butanoate	MS/RI	fruity, floral, green, winey	0.00	0.00	0.00	0.00	0.00	0.00	0.00	0.00	0.00	3563.06	0.00	1332.02	165.30	82.13	243.03	468.15	314.29	2424.03
276	1967	beta-ionone epoxide	MS/RI	fruity, sweet, berry, woody, violet, orris, powdery	331.78	702.61	1229.43	190.56	0.00	343.20	796.35	48.12	200.13	5661.50	869.31	1505.37	660.68	4101.97	797.89	1802.17	4478.29	5327.15
277	1968	butanoic acid, 2-methyl-, 2-phenylethyl ester	MS/RI	fruity, floral, green, sweet, waxy	0.00	0.00	0.00	0.00	0.00	0.00	0.00	0.00	5.72	1929.09	263.10	2940.92	95.77	0.00	0.00	0.00	0.00	1853.92
278	1971	1-(1H-pyrrol-2-yl)-ethanone	MS/RI	sweet, fruity, musty, cherry, nutty, wasabi, tea	0.00	0.00	48.68	23.63	15.94	15.20	313.18	0.00	48.31	3943.20	125.62	42.31	53.32	4694.79	907.86	1410.90	1661.39	444.49
279	1971	β-dihydroionol	MS/RI	woody, floral, amber	0.00	0.00	0.00	0.00	0.00	0.00	0.00	0.00	0.00	0.00	0.00	0.00	29.61	0.00	0.00	0.00	0.00	2667.22
280	1972	(Z)-jasmone	MS/RI/S/O	woody, bitter, tea, with a citrus and floral nuance	0.00	441.05	0.00	372.20	1303.26	2407.08	1141.35	88.03	5434.86	3864.04	1069.70	5924.99	792.13	834.95	805.90	615.68	733.15	14077.44

281	1972	dodecanol	MS/RI	fat, soapy	0.00	98.99	1287.76	0.00	0.00	0.00	0.00	0.00	0.00	0.00	0.00	0.00	0.00	0.00	0.00	0.00	0.00	0.00
282	1977	2H-pyran-2-one, tetrahydro-6-propyl-	MS/RI	coconut, sweet, creamy, lactonic and fruity	0.00	0.00	0.00	0.00	0.00	0.00	0.00	0.00	0.00	0.00	0.00	74.88	0.00	592.90	0.00	0.00	0.00	542.44
283	1992	phenol	MS/RI	phenol, plastic	344.58	82.77	175.93	434.66	0.00	80.38	99.79	40.99	103.25	772.72	124.67	196.20	129.22	970.67	312.78	448.19	874.16	596.05
284	2017	trans-nerolidol	MS/RI/S/O	green, floral, woody, fruity, citrus, melon	0.00	0.00	962.32	0.00	0.00	0.00	113.08	0.00	1297.37	37749.31	17922.78	61834.01	9415.46	6509.00	2678.10	4064.88	4044.98	31266.39
285	2021	perilla alcohol	MS/RI	woody, cumin, spicy, cardamom, waxy, floral, oily, earthy	0.00	0.00	0.00	0.00	0.00	6.58	0.00	0.00	0.00	84.31	0.00	33.64	0.00	114.91	40.14	66.99	91.51	82.98
286	2028	pyrrole-3-carboxaldehyde	MS/RI	musty, beefy, coffee	151.27	0.00	0.00	0.00	0.00	0.00	0.00	0.00	0.00	2067.33	54.06	6.39	0.00	2717.67	364.08	2050.21	373.94	310.95
287	2032	trans-cinnamaldehyde	MS/RI	sweet, spice, candy, cinnamon, red hots, warm	0.00	0.00	0.00	0.00	0.00	0.00	0.00	0.00	23.46	64.83	0.00	77.48	0.00	59.67	0.00	34.42	28.63	50.64
288	2034	phenylethyl valerate	MS/RI	fruity, rose, leaf	0.00	0.00	0.00	0.00	0.00	27.39	23.25	0.00	0.00	0.00	0.00	0.00	0.00	399.81	105.32	0.00	193.86	293.08
289	2042	dihydro-5-pentyl-2(3H)-furanone	MS/RI	coconut, peach	36.94	0.00	0.00	12.71	0.00	36.07	38.55	0.00	0.00	1240.69	227.12	66.90	53.67	882.04	130.33	532.93	863.00	1194.82
290	2043	furaneol	MS/RI/S/O	caramel	0.00	0.00	0.00	0.00	0.00	0.00	10.61	0.00	0.00	0.00	0.00	0.00	0.00	0.00	0.00	8.52	0.00	0.00
291	2049	o-methoxybenzoic acid methyl ester	MS/RI	herbal, floral, hyacinth, fruity, blackcurrant	0.00	0.00	0.00	0.00	0.00	5.63	0.00	0.00	0.00	0.00	0.00	18.99	0.00	10.04	0.00	0.00	72.88	45.56
292	2050	o-ethylphenol	MS/RI	phenolic	0.00	0.00	0.00	0.00	0.00	0.00	17.11	0.00	0.00	0.00	0.00	0.00	0.00	0.00	29.31	55.44	63.02	147.72
293	2050	nerolidol	MS/RI/S	floral, green, waxy, citrus, woody	271.10	0.00	162.53	17.30	0.00	0.00	0.00	0.00	0.00	194.36	116.27	796.51	0.00	188.51	0.00	0.00	0.00	559.86
294	2052	5-methyl-2-phenyl-2-hexenal	MS/RI	bitter cocoa, dark chocolate, aldehydic, coffee, nutty, honey, green	0.00	0.00	0.00	0.00	0.00	0.00	8.64	0.00	0.00	1297.75	0.00	0.00	0.00	176.78	1756.03	1580.99	1045.25	1065.79
295	2055	diphenyl ether	MS/RI	rose, metallic	172.49	22.54	149.06	35.42	24.76	176.10	141.28	26.47	272.83	213.22	0.00	52.48	51.72	134.96	231.71	269.05	388.96	285.83
296	2056	hexyl benzoate	MS/RI	sweet, green, fruity, balsamic, orchid, metallic,	0.00	0.00	0.00	0.00	0.00	0.00	0.00	0.00	0.00	668.39	123.29	101.59	0.00	237.94	144.01	0.00	230.65	715.02

				cilantro																		
297	2057	benzyl hexanoate	MS/RI	green, apricot, fruity, gardenia, jasmin	0.00	0.00	0.00	0.00	0.00	0.00	0.00	0.00	0.00	0.00	0.00	99.05	0.00	0.00	0.00	0.00	0.00	768.44
298	2058	cuminic alcohol	MS/RI	wood, herb	0.00	0.00	0.00	0.00	0.00	0.00	0.00	0.00	0.00	170.08	0.00	61.96	152.63	0.00	30.62	259.12	271.46	301.82
299	2067	4-methylphenol	MS/RI	medicine, phenol, smoke	0.00	0.00	21.05	51.15	0.00	8.49	22.65	0.00	0.00	637.26	11.51	0.00	0.00	657.38	174.51	273.58	480.09	474.29
300	2083	octanoic acid	MS/RI	sweat, cheese	0.00	0.00	0.00	0.00	0.00	0.00	8.67	0.00	0.00	1028.16	28.01	26.24	0.00	449.48	132.84	406.04	1020.38	1154.84
301	2093	cis-3-hexenyl benzoate	MS/RI	fatty, floral, green, fruity, spicy, woody	0.00	0.00	0.00	0.00	0.00	0.00	21.16	0.00	16.05	0.00	289.79	424.87	0.00	0.00	0.00	0.00	0.00	190.78
302	2103	γ-decalactone	MS/RI	peach, fat	0.00	0.00	0.00	0.00	0.00	0.00	0.00	0.00	0.00	0.00	12.27	39.65	0.00	0.00	0.00	0.00	34.24	53.70
303	2106	cedrol	MS/RI	woody, amber, floral, cedar, ambrette, musk	1110.86	1309.43	0.00	0.00	13.07	89.71	121.73	26.42	748.47	0.00	0.00	0.00	0.00	84.76	1867.00	0.00	3038.87	0.00
304	2115	m-cresol	MS/RI	fecal, plastic	0.00	0.00	0.00	42.21	0.00	6.09	0.00	23.21	0.00	0.00	0.00	0.00	100.46	0.00	0.00	0.00	0.00	0.00
305	2153	Thymol	MS/RI	phenolic, medicinal, woody, spicy	0.00	36.35	0.00	0.00	0.00	223.18	3.79	0.00	0.00	160.24	19.62	383.13	0.00	0.00	92.13	823.85	153.39	0.00
306	2160	2-phenylethyl hexanoate	MS/RI	sweet, waxy, floral, fruity	0.00	0.00	0.00	0.00	0.00	0.00	0.00	0.00	0.00	172.02	0.00	447.08	0.00	0.00	0.00	0.00	0.00	0.00
307	2175	τ-cadinol	MS/RI	balsam, earthy	0.00	0.00	0.00	0.00	0.00	59.18	0.00	14.92	0.00	0.00	62.07	0.00	0.00	0.00	181.33	883.59	298.06	0.00
308	2186	4-ethyl-phenol	MS/RI	phenolic, smoke, bacon and ham	0.00	0.00	37.93	2.76	0.00	0.00	0.00	0.00	0.00	94.51	4.76	0.00	0.00	319.10	0.00	0.00	0.00	0.00
309	2188	methyl 2-aminobenzoate	MS/RI	sweet, fruity, concord grape, with a musty and berry nuance	0.00	0.00	0.00	0.00	0.00	3.20	0.00	0.00	0.00	0.00	109.03	38.88	0.00	45.75	13.49	0.00	0.00	31.64
310	2189	carvacrol	MS/RI	spicy, herbal, phenolic, medicinal, woody	0.00	0.00	92.65	0.00	16.14	195.49	145.10	0.00	49.56	239.70	0.00	1097.66	1122.54	104.55	330.58	396.80	71.30	303.81
311	2190	δ-amylvalerolactone	MS/RI	coconut, creamy, fatty, buttery, milky, nutty, fruity	0.00	0.00	0.00	0.00	0.00	0.00	0.00	0.00	0.00	857.13	663.17	836.55	125.93	307.68	0.00	68.95	92.39	1228.74
312	2202	nonanoic acid	MS/RI	cheesy, fat	96.00	0.00	134.65	0.00	0.00	38.70	39.68	0.00	17.04	1146.29	22.42	29.98	0.00	221.75	244.02	457.89	780.36	1214.09
313	2221	α-cadinol	MS/RI	herb, wood	0.00	23.75	0.00	0.00	0.00	38.41	45.94	30.97	21.38	120.84	288.93	60.67	0.00	0.00	346.92	2085.39	397.60	174.56

314	2273	creamy lactone	MS/RI	creamy, fatty, coconut, peach and apricot	0.00	0.00	0.00	0.00	0.00	0.00	0.00	0.00	5.51	4541.90	3143.94	12746.83	2661.66	1893.83	77.35	152.77	240.63	8009.21
315	2325	(2,6,6-trimethyl-2-hydroxycyclohexylidene) acetic acid lactone	MS/RI	fruity, apricot, woody, mango, berry, cherry, grape	0.00	237.11	521.12	80.94	0.00	73.19	165.85	0.00	56.86	0.00	0.00	0.00	0.00	0.00	570.80	0.00	0.00	0.00
316	2332	methyl jasmonate	MS/RI	floral, magnolia, fruity, green, jasmin, waxy, kiwi, melon	0.00	0.00	0.00	0.00	0.00	0.00	0.00	0.00	18.29	0.00	0.00	449.49	174.87	0.00	0.00	0.00	0.00	231.46
317	2350	trans-farnesol	MS/RI	floral sweet	0.00	0.00	0.00	0.00	0.00	0.00	0.00	0.00	13.18	0.00	0.00	140.12	0.00	95.64	27.49	0.00	0.00	0.00
318	2441	Indole	MS/RI/S/O	fecal, perfumey	0.00	613.15	544.02	651.85	543.97	360.50	154.50	91.95	479.00	27578.99	0.00	39547.13	19184.91	5199.40	1222.19	2183.80	939.07	3594.80
